# Effect of influenza vaccination on the outcomes of hospitalization for kidney disease in a geriatric population: A propensity-score matched study

**DOI:** 10.1371/journal.pone.0262420

**Published:** 2022-01-25

**Authors:** Chien-Chang Liao, Ying-Hsuan Tai, Chun-Chieh Yeh, Yung-Ho Hsu, Ta-Liang Chen, Yih-Giun Cherng

**Affiliations:** 1 Department of Anesthesiology, Taipei Medical University Hospital, Taipei, Taiwan; 2 Anesthesiology and Health Policy Research Center, Taipei Medical University Hospital, Taipei, Taiwan; 3 Department of Anesthesiology, School of Medicine, College of Medicine, Taipei Medical University, Taipei, Taiwan; 4 Research Center of Big Data and Meta-Analysis, Wan Fang Hospital, Taipei Medical University, Taipei, Taiwan; 5 School of Chinese Medicine, College of Chinese Medicine, China Medical University, Taichung, Taiwan; 6 Department of Anesthesiology, Shuang Ho Hospital, Taipei Medical University, New Taipei City, Taiwan; 7 Department of Surgery, China Medical University Hospital, China Medical University, Taichung, Taiwan; 8 Department of Surgery, University of Illinois, Chicago, Illinois, United States of America; 9 Department of Nephrology, Shuan Ho Hospital, Taipei Medical University, New Taipei City, Taiwan; 10 Department of Anesthesiology, Wan Fang Hospital, Taipei Medical University, Taipei, Taiwan; Nazarbayev University School of Medicine, KAZAKHSTAN

## Abstract

**Background and aims:**

The effects of influenza vaccination (IV) on the outcomes of patients with kidney disease (KD) are not completely understood. We aimed to evaluate and compare the outcomes during admission of KD between elderly patients who did or did not receive an IV within the previous 12 months.

**Methods:**

We used health insurance research data in Taiwan and conducted a population-based cohort study that included 22,590 older people aged ≥ 65 years who were hospitalized for KD in 2008–2013. We performed propensity score matching (case-control ratio 1:1) to select 4386 eligible IV recipients and 4386 nonrecipient controls for comparison. The adjusted odds ratios (ORs) with 95% confidence intervals (CIs) of IV associated with complications and mortality during KD admission were calculated using multivariable logistic regression analyses.

**Results:**

During hospitalization for KD, IV was significantly associated with lower risks of 30-day in-hospital mortality (OR 0.56, 95% CI 0.39–0.82), septicemia (OR 0.77, 95% CI 0.68–0.87), and intensive care (OR 0.85, 95% CI 0.75–0.96). Additionally, IV recipients had a shorter length of hospital stay and lower medical expenditure than nonrecipients. Subgroup analyses further showed that the association of IV with reduced adverse events was confined to patients aged ≥ 75 years.

**Conclusions:**

Previous IV was associated with reduced risks of complications and mortality and in elderly patients hospitalized for KD. We raised the possibility and suggested the need to promote IV for this susceptible population of patients with KD.

## Introduction

Kidney disease (KD) is a leading cause of premature mortality and disability, affecting approximately 700 million people and leading to 1.2 million deaths annually worldwide [1, 2]. Patients with KD are susceptible to infection and related complications due to their compromised immune function and exaggerated inflammatory response to pathogens [3, 4]. A graded association between a reduced glomerular filtration rate and mortality risk has been observed in patients with chronic renal insufficiency [5]. Among people with KD, older patients are at a higher risk of mortality than younger patients [6]. However, few therapeutic strategies have been proven shown to be effective in improving clinical outcomes for patients with KD [7, 8].

Influenza is a common respiratory infection and is considered an important burden on health and society worldwide [9]. It has a substantial impact on the general population and may cause serious complications, particularly in the elderly and patients with chronic diseases [10, 11]. Influenza causes higher rates of mortality and morbidity in patients with KD than in individuals without KD [11, 12].

Influenza vaccination (IV) has been shown to be effective in reducing hospitalizations for influenza, pneumonia, cardiovascular events, and all-cause mortality among the elderly population [13–16] or patients with stroke [17] and pulmonary diseases [18, 19]. Several studies have investigated the beneficial effects of IV on reducing infection and other complications in patients with KD [20–24]. However, the relationship between IV and outcomes of KD has not yet been completely clarified due to some limitations of previous studies, including the inconsistent definition of old age [22, 23], inadequate control for confounding factors [22, 23], the lack of a comprehensive evaluation of critical outcomes [22, 23], and a focus on specific kidney diseases [20, 21, 24]. In addition, most previous studies evaluated the long-term outcomes (one year after vaccination) of patients with KD [20–24]. Therefore, researchers have not clearly determined whether IV is associated with short-term mortality and morbidity during hospitalization for KD. Accordingly, we conducted a population-based cohort study to evaluate the association of IV with subsequent mortality and morbidity among elderly people hospitalized for KD.

## Methods

### Data source

In this study, we analyzed the research database of health insurance in Taiwan. Insurance programs have been implemented since 1995 and cover more than 99% of the Taiwanese population. The available information of this research database includes age, sex, birth date, diagnoses, examinations, prescriptions, treatments, and medical expenditures for outpatient and inpatient medical services. The reliability of this database has been well accepted by important international scientific journals [14, 17–19, 21–24]. Information on beneficiaries was scrambled to protect personal privacy and all data were fully anonymized before we accessed them. Our research team accessed the research database to obtain the retrospective data used in this study in December 5, 2017. This study was approved by the institutional review board of Taipei Medical University that also waived the need for informed consent of this study (TMU-JIRB-201509050; TMU-JIRB-201505055; TMU-JIRB-2022).

### Study design

**Fig 1:**
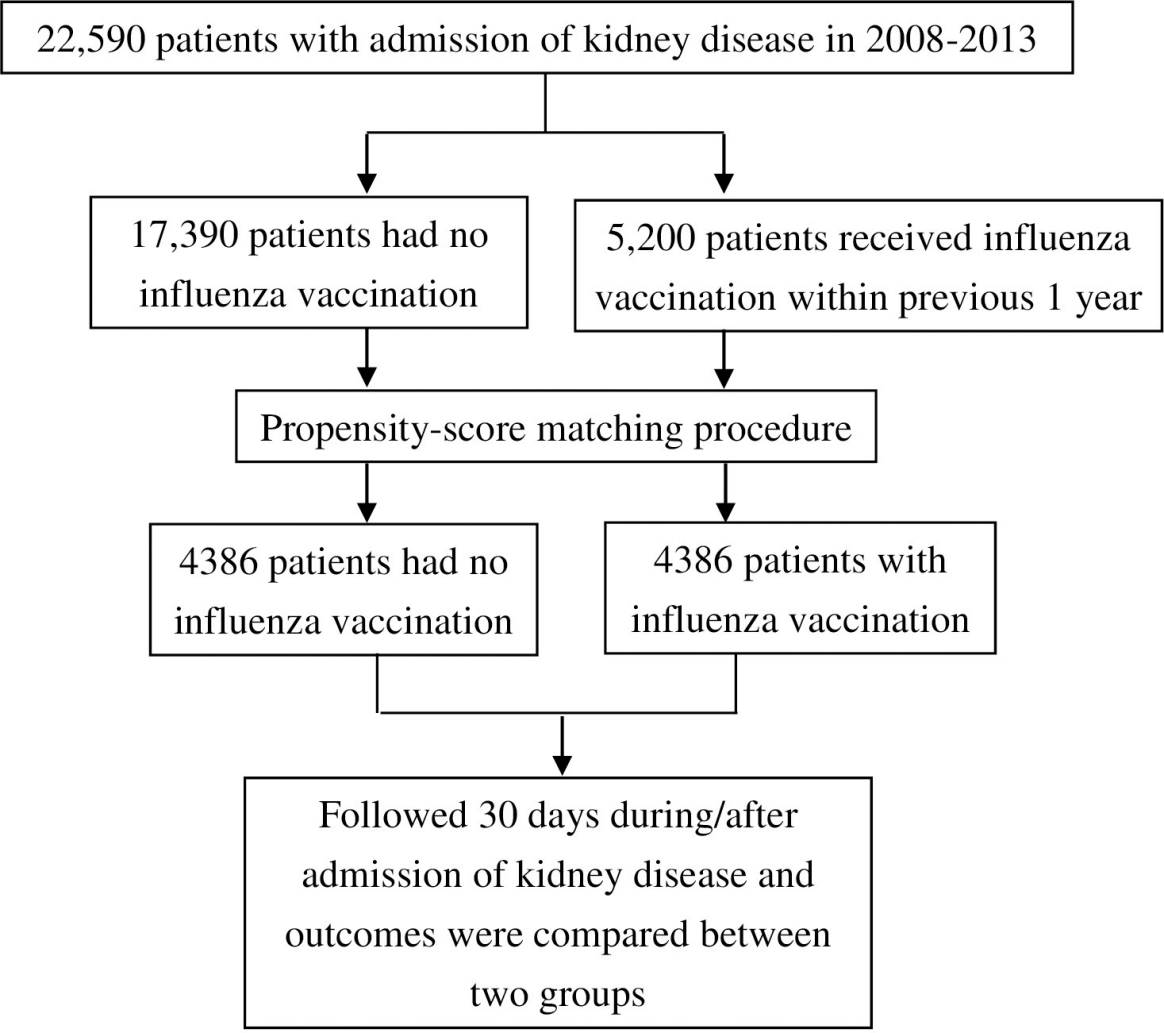
Selection process of study participants.

In a nested retrospective cohort study, we initially included 22,590 patients aged more than 65 years who were hospitalized for KD during 2008–2013. We selected 5,200 patients who had received IV within 12 months before the index KD admission. Each patient with KD who received IV was matched with an unvaccinated patient with KD using the propensity score matching procedure to balance sociodemographic characteristics (age, sex, and low income), types of kidney disease, and coexisting medical conditions. The selection process of study participants was shown in Fig 1. In this study, the inclusion criteria of KD patients are based on the International Classification of Diseases, Ninth Revision, Clinical Modification (ICD-9-CM) codes (included 580–588, 590, and 591) and physicians’ primary diagnosis of hospitalization.

### Measures and definitions

In this study, we defined the short-term outcomes as identifying complications and mortality during the index hospitalization. The low-income status of patients was defined as qualifying for waived medical copayment, as verified by the Ministry of Health and Welfare, Taiwan. The ICD-9-CM and physicians’ diagnoses were used to define coexisting medical conditions and complications during hospitalization for KD. The coexisting medical conditions were determined from medical claims in the 24-month period before the index admission, including hypertension (ICD-9-CM 401–405), diabetes (ICD-9-CM 250), mental disorders (ICD-9-CM 290–319), ischemic heart disease (ICD-9-CM 410–414), chronic obstructive pulmonary disease (ICD-9-CM 491, 492, and 496), heart failure (ICD-9-CM 428), hyperlipidemia (ICD-9-CM 272.0, 272.1, 272.2, and 272.4), Parkinson’s disease (ICD-9-CM 332), and liver cirrhosis (ICD-9-CM 571.2, 571.5, and 571.6). Renal dialysis was defined by administration codes (D8 and D9). Complications during KD admission included pneumonia (ICD-9-CM 480–486), septicemia (ICD-9-CM 038 and 998.5), urinary tract infection (ICD-9-CM 599.0), acute myocardial infarction (ICD-9-CM 410), and stroke (ICD-9-CM 430–437). We also considered admission to the intensive care unit, the length of the hospital stay, and medical expenditures during KD admission as study outcomes. We compared the outcomes between patients with or without prior IV.

### Statistical analysis

The frequency matching and propensity-score matching were used to conduct the current matching procedure. We performed the propensity score matching analysis to balance the distribution of baseline demographic and clinical characteristics between patients with and without IV. A nonparsimonious multivariable logistic regression model was used to estimate a propensity score for patients with KD who received IV. Based on previous related studies [14, 17], the covariates included in this model were chosen based on clinical significance, including age, sex, low income, type of KD, hypertension, mental disorders, diabetes, chronic obstructive pulmonary disease, ischemic heart disease, heart failure, hyperlipidemia, liver cirrhosis, Parkinson’s disease, and renal dialysis. We matched patients with KD who received IV to non-IV patients using a greedy matching algorithm (without replacement) with a caliper width of 0.2 SD of the log odds of the estimated propensity score. We used Statistical Analysis System software version 9.1 (SAS Institute Inc, Cary, NC) to perform data analyses.

We used frequencies (percentages) and chi-square tests to analyze and compare baseline categorical variables between patients with KD with and without IV. We analyzed continuous variables using t tests and by calculating the means ± standard deviations. The adjusted odds ratios (ORs) and 95% confidence intervals (CIs) of IV associated with mortality and morbidity during KD admission were analyzed with multivariable logistic regression analyses. Additional analyses stratified by age, sex, and number of medical conditions were performed to examine KD outcomes in patients with and without IV within these strata.

## Results

The unmatched baseline characteristics of patients with KD who did or did not receive IV are shown in Table 1 (N = 22,590). After matching (Table 2), no differences in the baseline characteristics were observed between patients with KD who did or did not receive without IV (N = 8772).

**Table 1:** Characteristics of hospitalized kidney disease patients with and without influenza vaccination (before matching).

	No IV (N = 17,390)		IV (N = 5,200)		*p*-value
Sex	n	(%)	n	(%)	<0.0001
Female	9983	(57.4)	2770	(53.3)	
Male	7407	(42.6)	2430	(46.7)	
Age, years					<0.0001
65–69	4323	(24.9)	736	(14.2)	
70–74	4155	(23.9)	1282	(24.7)	
75–79	3791	(21.8)	1397	(26.9)	
80–84	2987	(17.2)	1086	(20.9)	
≥ 85	2134	(12.3)	699	(13.4)	
Low income					0.7889
No	16927	(97.3)	5058	(97.3)	
Yes	463	(2.7)	142	(2.7)	
Medical conditions					
Hypertension	7554	(43.4)	2477	(47.6)	<0.0001
Diabetes	6108	(35.1)	1684	(32.4)	0.0003
Mental disorders	3839	(22.1)	1298	(25.0)	<0.0001
COPD	2174	(12.5)	901	(17.3)	<0.0001
Ischemic heart disease	3329	(19.1)	1099	(21.1)	0.0015
Heart failure	2103	(12.1)	567	(10.9)	0.0198
Parkinson’s disease	771	(4.4)	248	(4.8)	0.3062
Liver cirrhosis	544	(3.1)	173	(3.3)	0.4733
Hyperlipidemia	853	(4.9)	341	(6.6)	<0.0001
Types of kidney disease					<0.0001
Acute glomerulonephritis	81	(0.5)	24	(0.5)	
Nephrotic syndrome	221	(1.3)	79	(1.5)	
Chronic glomerulonephritis	124	(0.7)	46	(0.9)	
Nephritis and nephropathy	80	(0.5)	31	(0.6)	
Acute kidney failure	5844	(33.6)	1639	(31.5)	
Chronic kidney disease	2888	(16.6)	763	(14.7)	
Renal failure, unspecified	163	(0.9)	42	(0.8)	
Renal sclerosis, unspecified	10	(0.1)	7	(0.1)	
Disorders from impaired renal function	489	(2.8)	182	(3.5)	
Infections of kidney	5661	(32.6)	1812	(34.9)	
Hydronephrosis	1827	(10.5)	575	(11.1)	

COPD, chronic obstructive pulmonary disease; IV, influenza vaccination.

**Table 2:** Characteristics of hospitalized kidney disease patients with and without influenza vaccination (after propensity-score matching).

	No IV (N = 4386)		IV (N = 4386)		*p*-value
Sex	n	(%)	n	(%)	1.0000
Female	2396	(54.6)	2396	(54.6)	
Male	1990	(45.4)	1990	(45.4)	
Age, years					1.0000
65–69	645	(14.7)	645	(14.7)	
70–74	1095	(25.0)	1095	(25.0)	
75–79	1201	(27.4)	1201	(27.4)	
80–84	899	(20.5)	899	(20.5)	
≥ 85	546	(12.5)	546	(12.5)	
Low income					1.0000
No	4340	(99.0)	4340	(99.0)	
Yes	46	(1.0)	46	(1.0)	
Medical conditions					
Hypertension	2033	(46.4)	2033	(46.4)	1.0000
Diabetes	1386	(31.6)	1386	(31.6)	1.0000
Mental disorders	932	(21.3)	932	(21.3)	1.0000
Ischemic heart disease	769	(17.5)	769	(17.5)	1.0000
COPD	563	(12.8)	563	(12.8)	1.0000
Heart failure	362	(8.3)	362	(8.3)	1.0000
Hyperlipidemia	170	(3.9)	170	(3.9)	1.0000
Parkinson’s disease	99	(2.3)	99	(2.3)	1.0000
Liver cirrhosis	85	(1.9)	85	(1.9)	1.0000
Types of kidney disease					1.0000
Acute glomerulonephritis	9	(0.2)	9	(0.2)	
Nephrotic syndrome	40	(0.9)	40	(0.9)	
Chronic glomerulonephritis	19	(0.4)	19	(0.4)	
Nephritis and nephropathy	11	(0.3)	11	(0.3)	
Acute kidney failure	1451	(33.1)	1451	(33.1)	
Chronic kidney disease	637	(14.5)	637	(14.5)	
Renal failure, unspecified	22	(0.5)	22	(0.5)	
Renal sclerosis, unspecified	2	(0.1)	2	(0.1)	
Disorders from impaired renal function	132	(3.0)	132	(3.0)	
Infections of kidney	1590	(36.3)	1590	(36.3)	
Hydronephrosis	473	(10.8)	473	(10.8)	

COPD, chronic obstructive pulmonary disease; IV, influenza vaccination.

Before matching by propensity score (S1 Table), IV was associated with mortality (OR 0.66, 95% CI 0.50–0.88), septicemia (OR 0.83, 95% CI 0.76–0.92), and intensive care (OR 0.87, 95% CI 0.79–0.95). After propensity-score matching (Table 3), patients with KD and a previous IV had lower risks of septicemia (OR 0.77, 95% CI 0.68–0.87), need for intensive care (OR 0.85, 95% CI 0.75–0.96), and in-hospital mortality (OR 0.56, 95% CI 0.39–0.82), compared with the control group. The length of hospital stay (10.0±11.5 vs. 11.4±15.1 days, *p*<0.0001) and medical expenditures (1973±3157 vs. 2294±3580 US dollars, *p*<0.0001) were both lower in patients with KD who received IV than in non-IV controls. After propensity-score matching, the linear regression analyses showed that IV was associated with reduced length of hospital stay (beta = -1.4, *p*<0.0001) and medical expenditures (beta = -320.8, *p*<0.0001).

**Table 3:** Adverse outcomes of kidney disease admissions for patients with and without influenza vaccination (after propensity-score matching).

	No IV (N = 4,386)		IV (N = 4,386)		Risk of outcomes	
Outcomes	Events	%	Event	%	OR	(95% CI)*
30-day in-hospital mortality	77	1.8	44	1.0	0.56	(0.39–0.82)
Pneumonia	414	9.4	381	8.7	0.91	(0.79–1.06)
Septicemia	649	14.8	517	11.8	0.77	(0.68–0.87)
Urinary tract infection	1183	27.0	1189	27.1	1.01	(0.92–1.11)
Stroke	267	6.1	238	5.4	0.88	(0.74–1.06)
Acute myocardial infarction	29	0.7	34	0.8	1.18	(0.71–1.94)
ICU stay	619	14.1	542	12.4	0.85	(0.75–0.96)
Medical expenditure, US dollars†	2294±3580		1973±3157		*p*<0.0001	
Medical expenditure, US dollars ‡	1124±1701		1004±1573		*p*<0.0001	
Length of hospital stay, days†	11.4±15.1		10.0±11.5		*p*<0.0001	
Length of hospital stay, days‡	7.0±9.0		7.0±8.0		*p*<0.0001	

CI, confidence interval; ICU, intensive care unit; IV, influenza vaccination; OR, odds ratio.

*Adjusted for all covariates listed in Table 1.

†Mean±SD; IV associated with medical expenditure (beta = -320.8, *p*<0.0001) and length of hospital stay (beta = -1.4, *p*<0.0001) in the linear regression models.

‡Median±interquartile range

After propensity-score matching, the stratified analysis (Table 4) showed that receiving IV was associated with reduced adverse events during KD admission (including septicemia, intensive care, and mortality) among men (OR 0.72, 95% CI 0.60–0.87), women (OR 0.79, 95% CI 0.67–0.93), and patients aged 75–79 years (OR 0.78, 95% CI 0.62–0.98), 80–84 years (OR 0.67, 95% CI 0.51–0.87), and ≥ 85 years (OR 0.66, 95% CI 0.49–0.90). In addition, IV was associated with adverse events in patients with one medical condition (OR 0.61, 95% CI 0.50–0.74).

**Table 4:** The stratified analysis for the risk of adverse events during hospitalization of kidney disease associated with influenza vaccination (after propensity-score matching).

			Adverse events*			
		n	Events	Rate, %	OR	(95% CI)†
Female	No IV	2396	382	15.9	1.00	(reference)
	IV	2396	313	13.1	0.79	(0.67–0.93)
Male	No IV	1990	308	15.5	1.00	(reference)
	IV	1990	234	11.8	0.72	(0.60–0.87)
Age 65–69 years	No IV	645	74	11.5	1.00	(reference)
	IV	645	73	11.3	0.98	(0.70–1.39)
Age 70–74 years	No IV	1095	146	13.3	1.00	(reference)
	IV	1095	120	11.0	0.80	(0.62–1.03)
Age 75–79 years	No IV	1201	193	16.1	1.00	(reference)
	IV	1201	156	13.0	0.78	(0.62–0.98)
Age 80–84 years	No IV	899	155	17.2	1.00	(reference)
	IV	899	110	12.2	0.67	(0.51–0.87)
Age ≥ 85 years	No IV	546	122	22.3	1.00	(reference)
	IV	546	88	16.1	0.66	(0.49–0.90)
0 medical condition	No IV	790	104	13.2	1.00	(reference)
	IV	790	89	11.3	0.84	(0.62–1.13)
1 medical condition	No IV	1682	285	16.9	1.00	(reference)
	IV	1682	186	11.1	0.61	(0.50–0.74)
2 medical conditions	No IV	1227	193	15.7	1.00	(reference)
	IV	1227	167	13.6	0.84	(0.67–1.06)
≥ 3 medical conditions	No IV	687	108	15.7	1.00	(reference)
	IV	687	105	15.3	0.97	(0.72–1.30)

CI, confidence interval; IV, influenza vaccination; OR, odds ratio.

*Adverse events included with 30-day in-hospital mortality, septicemia.

†Adjusted for all covariates listed in Table 1.

## Discussion

As shown in the present study, elderly patients receiving IV within the previous 12 months had reduced risks of septicemia, intensive care, and mortality during hospitalizations for KD. A shorter length of hospital stay and lower medical expenditures were also noted in IV users compared to non-IV patients during KD admission. The beneficial effect of IV was especially significant in patients with KD aged ≥ 75 years. To our knowledge, this study is the first to focus on the short-term outcomes during KD admission, which provides an important clinical implication in the context of kidney failure.

Few studies have investigated the relationship between IV and KD outcomes [20–24]. Compared with previous studies [20–24], we included a variety of kidney diseases and performed a comprehensive evaluation of critical outcomes in patients with KD to increase the generalizability of our results. A prior IV was associated with lower risks of all-cause mortality, septicemia, and need for intensive care, consistent with previous studies [20–24]. Although the rates of pneumonia and stroke were lower in the vaccine cohort than in the control cohort, the association did not reach statistical significance, in contrast to some studies [13, 16, 17, 21]. This discrepancy is possibly attributable to our relatively shorter follow-up period compared with previous studies [13, 16, 21]. IV prior to hospitalization reduces the risks of mortality and readmission among geriatric populations admitted with pneumonia [25, 26]. Based on our results, the protective effect of IV may be generalizable to hospitalized patients with KD, irrespective of the types of kidney disease and different medical conditions. Notably, our stratified analyses showed that the association between IV and a reduced number of adverse events was stronger in the subgroup of age ≥ 75 years, consistent with some other studies [22, 23].

We proposed the following explanations for the observed phenomena in this study. First, patients with influenza virus infection are susceptible to a secondary bacterial infection, which contributes to serious complications, such as mortality and the need for intensive care [27]. IV may protect against secondary bacterial infections by preventing influenza infection and improving the in-hospital outcome during KD admission. Second, previous studies suggested that IV reduces the risk of hospitalizations for influenza, pneumonia, cardiovascular diseases, and all-cause mortality [13–19, 22, 23, 25, 26]. Influenza infection might exacerbate coexisting diseases, especially in fragile patients with KD. The health condition of vaccine recipients may have been better than nonrecipients and hence improved in-hospital outcomes of KD hospitalization. Third, hospitalized patients are susceptible to nosocomial influenza and pneumonia [16, 28]. A prior IV might protect against possible nosocomial infections and thereby reduce overall mortality. Fourth, we found that IV was associated with fewer adverse events in patients aged ≥ 75 years but not < 75 years. Based on this finding, older people are more vulnerable to influenza infection and associated complications than younger people [29]. Therefore, older patients might have benefitted more from IV than their younger counterparts in this study. Finally, patients receiving IV annually might have higher levels of health consciousness, ambulatory independence, and support from family members and social networks than nonrecipients. Patients’ knowledge, attitudes, and practice of health behaviors and socioeconomic factors may influence the outcomes of patients with KD [30, 31].

Our results indicate an urgent need to promote IV for elderly patients with KD. However, the administration of vaccines to this vulnerable population is challenging. Antibody responses to IV, measured as seroconversion and seroprotection, decrease with increasing age [32]. The immunogenicity of the influenza vaccine is also attenuated in patients with kidney failure [33]. Current guidelines highly recommend the annual administration of seasonal inactivated influenza vaccines to adults with KD [34]. Among geriatric patients with KD, a high-dose trivalent influenza vaccine may be safe and immunogenic [35, 36]. In addition, an MF59-adjuvanted influenza vaccine exhibited better immunogenicity in patients with end-stage renal disease in a previous study [37]. Regarding the optimal influenza vaccine schedules for patients with KD, sufficient scientific evidence from large-scale randomized trials is unavailable [35, 38].

Our study has some limitations. First, distinct serotypes of seasonal influenza vaccines and vaccine adjuvants may activate different levels of immunogenic reactions [37, 39]. Although the present study was unable to standardize types of vaccines, we used the stated serotypes of vaccines used each year. Second, our database did not contain information on host-related factors that may affect influenza vaccine responsiveness, such as preexisting immunity and genetic polymorphisms [40]. Third, baseline kidney function and the duration of KD for each subject was unknown due to data unavailability. Fourth, the cause of death was unclear, and a causal relationship between vaccination and a reduced mortality rate is difficult to infer. Fifth, we did not measure markers of inflammatory and immune responses and therefore were unable to elucidate the biological mechanism underlying the results of our statistical analyses. Finally, we admitted that residual confounding possibly existed in this study since we were unable to control all potential confounding factors in the current analysis.

In conclusion, prior receipt of IV was significantly associated with reduced mortality, septicemia, and need for intensive care in geriatric patients hospitalized for KD. Additionally, vaccine recipients had a shorter length of hospitalization and lower medical expenditures than nonrecipients. The protective effect of IV was especially significant in patients aged ≥ 75 years. These results provide evidence to promote the delivery of influenza vaccines to patients with KD as a prophylactic measure to protect against adverse events.

## Supporting information

S1 Table Adverse outcomes of kidney disease admissions for patients with and without influenza vaccination (before matching). (DOCX)

## List of abbreviations

ICD-9-CM: International Classification of Diseases, 9th Revision, Clinical Modification
CI: confidence interval
IV: influenza vaccination
KD: kidney disease
OR: odds ratio
