# Supplementary material for: Effect of influenza vaccination on the outcomes of hospitalization for kidney disease in a geriatric population: A propensity-score matched study
Source: PLoS One. 2022 Jan 25;17(1):e0262420. doi: 10.1371/journal.pone.0262420 (PMC8789174; doi:10.1371/journal.pone.0262420)
Supplement: S1 Table — (DOCX) [file pone.0262420.s001.docx]

| **Table S1. Adverse outcomes of kidney disease admissions for patients with and without influenza vaccination (before matching)** | | | | | | |
| --- | --- | --- | --- | --- | --- | --- |
|  | No IV (N=17,390) | | IV (N=5,200) | | Risk of outcomes | |
| Outcomes | Events | % | Event | % | OR | (95% CI)* |
| 30-day in-hospital mortality | 282 | 1.6 | 58 | 1.1 | 0.66 | (0.50-0.88) |
| Pneumonia | 1667 | 9.6 | 461 | 8.9 | 0.90 | (0.81-1.01) |
| Septicemia | 2438 | 14.0 | 628 | 12.1 | 0.83 | (0.76-0.92) |
| Urinary tract infection | 4745 | 27.3 | 1392 | 26.8 | 0.97 | (0.90-1.04) |
| Stroke | 1028 | 5.9 | 318 | 6.1 | 1.00 | (0.87-1.14) |
| Acute myocardial infarction | 117 | 0.7 | 40 | 0.8 | 1.15 | (0.80-1.66) |
| ICU stay | 2583 | 14.9 | 657 | 12.6 | 0.87 | (0.79-0.95) |
| Medical expenditure† | 2276±3439 | | 1962±3114 | | *p*<0.0001 | |
| Length of hospital stay, days† | 11.5±15.1 | | 10.0±11.7 | | *p*<0.0001 | |
| CI, confidence interval; ICU, intensive care unit; IV, influenza vaccination; OR, odds ratio.  *Adjusted for all covariates listed in Table 1.  †Mean±SD; IV associated with medical expenditure (beta=-278, *p*<0.0001) and length of hospital stay (beta=-1.5, *p*<0.0001) in the linear regression models. | | | | | | |
